# The Musite open-source framework for phosphorylation-site prediction

**DOI:** 10.1186/1471-2105-11-S12-S9

**Published:** 2010-12-21

**Authors:** Jianjiong Gao, Dong Xu

**Affiliations:** 1 Department of Computer Science, C.S. Bond Life Sciences Center, University of Missouri, Columbia, Missouri 65211, USA

## Abstract

**Background:**

With the rapid accumulation of phosphoproteomics data, phosphorylation-site prediction is becoming an increasingly active research area. More than a dozen phosphorylation-site prediction tools have been released in the past decade. However, there is currently no open-source framework specifically designed for phosphorylation-site prediction except Musite.

**Results:**

Here we present the Musite open-source framework for building applications to perform machine learning based phosphorylation-site prediction. Musite was implemented with six modules loosely coupled with each other. With its well-designed Java application programming interface (API), Musite can be easily extended to integrate various sources of biological evidence for phosphorylation-site prediction.

**Conclusions:**

Released under the GNU GPL open source license, Musite provides an open and extensible framework for phosphorylation-site prediction. The software with its source code is available at http://musite.sourceforge.net.

## Background

Protein phosphorylation is one of the most studied posttranslational modifications (PTM). It is an important regulatory event, playing essential roles in many aspects of cell life [1]. The protein phosphorylation data have increased rapidly in the past decade, thanks to the high-throughput studies [2-6] and web resources [7-15]. However, experimental identification of phosphorylation sites is still an expensive and time-consuming task. Computational prediction of phosphorylation sites provides a useful alternative approach for phosphorylation site identification, and hence has become an active research area.

Musite [16] was the first open-source framework specifically designed for phosphorylation-site prediction that meets the Open Source Initiative (OSI) Open Standards Requirement (http://www.opensource.org/osr-intro). There are more than a dozen phosphorylation-site prediction tools available before Musite, including DISPHOS [17], NetPhos [18], scan-x [19], ScanSite [20], NetPhosK [21], GPS [22], KinasePhos [23], Predikin [24], CRPhos [25], AutoMotif [26], pkaPS [27], PhoScan [28], PredPhospho [29], and NetPhorest [30]. Machine learning techniques were adopted in most of these tools. Although there are general open-source machine learning frameworks such as BioWeka [31] that support feature extraction from local sequence properties, there was no open-source framework specifically designed for phosphorylation-site prediction before Musite.

We have developed Musite, an open-source software tool for large-scale prediction of both general and kinase-specific phosphorylation-site prediction. In [16], we introduced its methodology and validated it by applying to several proteomes and comparing it to other tools. In this paper, we will describe the underlying open-source software framework of Musite.

## Implementation

### Machine learning based framework

**Figure 1:**
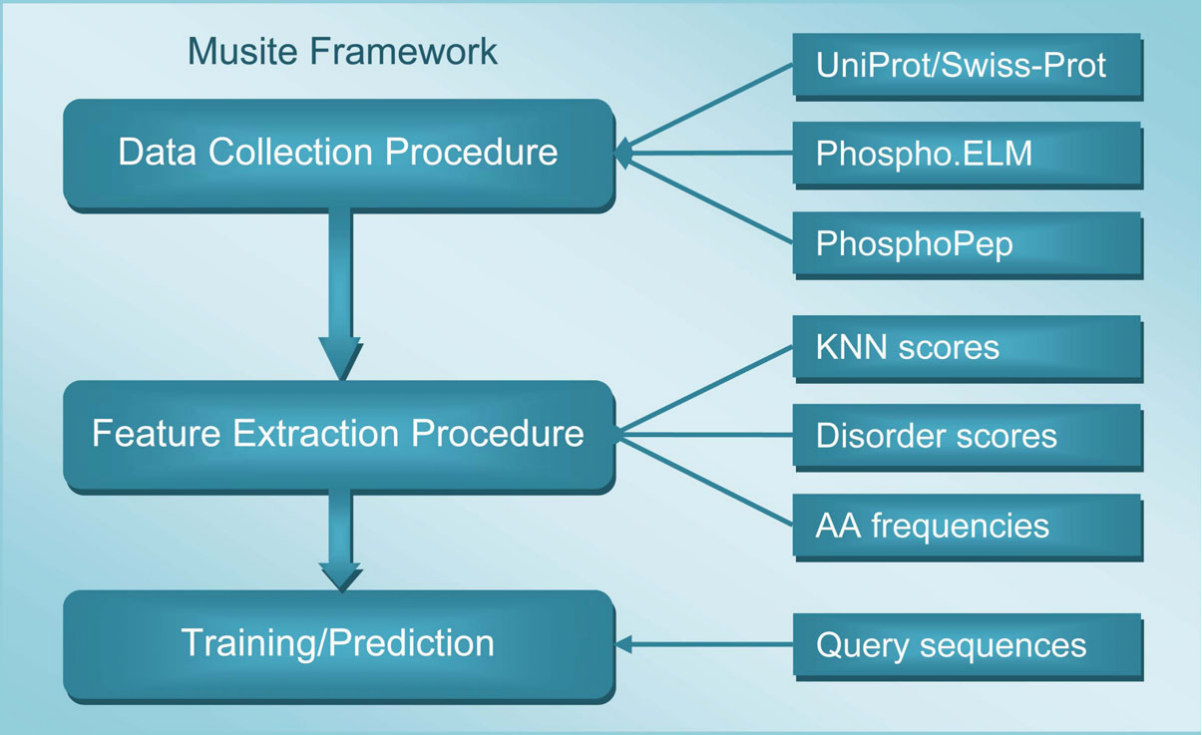
**Overall Musite Framework.** The data collection procedure collects phosphorylation data from various sources. The feature extraction procedure extracts multiple features for prediction model training. The training/prediction procedure trains prediction models and makes predictions for new query sequences. All procedures are extensible, for example, more data sources can be added and more types of features can be extracted.

In Musite, we formulated the problem of phosphorylation-site prediction as a machine learning problem, specifically a binary classification problem, i.e., protein residues can be classified into two categories: phosphorylation sites and non-phosphorylation sites. The Musite framework is an implementation to solve this machine learning problem, which consists of three main procedures: data collection, feature extraction, and training/prediction, as shown in Figure 1.

The data collection procedure is designed for collecting phosphorylation data from various sources and converting them into formats that Musite accepts. For example, phosphorylation sites can be easily retrieved from UniProt/Swiss-Prot using the utility of converting UniProt XML to Musite XML. Musite also has functionalities of merging phosphorylation annotations from different sources and building non-redundant datasets.

The feature extraction procedure extracts features from the collected data to characterize patterns of phosphorylation sites. To date, three sets of features have been integrated in Musite, namely *k*-nearest neighbor (KNN) scores, protein disorder scores, and amino acid (AA) frequencies [16]. We are currently in the process of evaluating more features, such as solvent accessibility and secondary structure information. A feature will be integrated after evaluation if it meets the following criteria: 1) it is relevant to the biological context, i.e., it is related to protein phosphorylation; 2) it helps to improve prediction performance; and 3) it is computationally feasible for large-scale predictions.

In the training and prediction procedure, binary classifiers are trained using the features extracted from training data. The trained classifiers can then be used to predict phosphorylation sites in users’ query protein sequences. We have integrated a support vector machine (SVM) classifier, and we also implemented a bootstrap classifier and a boosting classifier, which were combined to implement a bootstrap aggregating procedure.

Other utilities were also provided to assist phosphorylation-site prediction and analysis in Musite, including prediction model management, customized model training, specificity estimation, filtering, statistics, etc.

### Java API

**Figure 2:**
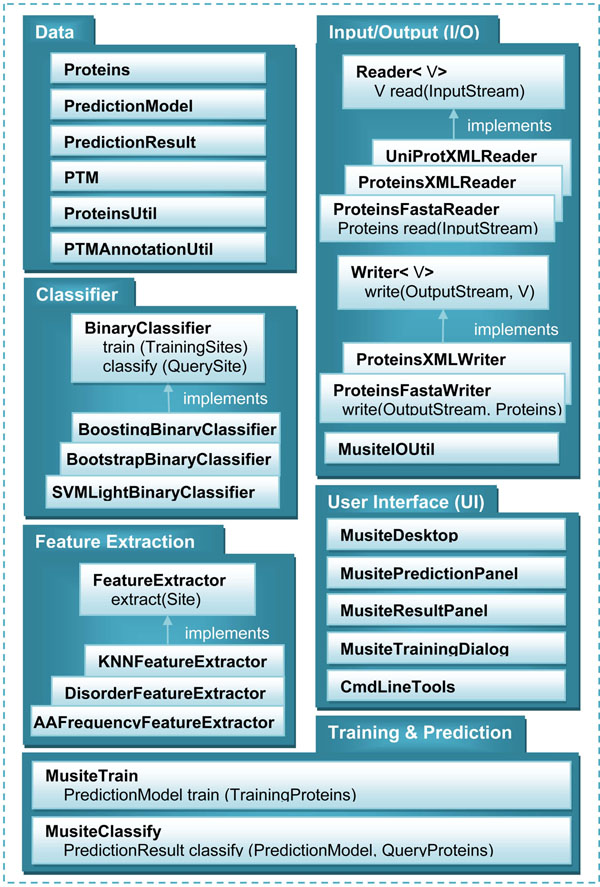
**Simplified UML diagram of Musite.** Musite architecture contains six modules loosely coupled with each other. The data module defines the core data structure. The classifier module contains a set of binary classifiers. The feature extraction module defines the features to be extracted from data and used in classifiers. The training and prediction module defines the machine learning procedure. The I/O module provides utilities for reading/writing different types of files and converting between them. The UI module provides users with a biologist-friendly GUI.

Musite is written in Java and released under the GNU GPL open source license.Figure 2 illustrates its overall architecture and application programming interface (API). Musite architecture contains six modules that are loosely coupled with each other.

The data module defines the core data structure in Musite, representing protein information, posttranslational modification, prediction model, and prediction result, etc. This module also contains several utility classes, for example, PTMAnnotationUtil is a class for annotating phosphorylation and other PTM sites in proteins. All other modules are dependent on this module.

The classifier module, feature extraction module, and training and prediction module form the core modules of machine learning. The classifier module contains a set of binary classifiers. We have incorporated SVM^light^[32] and implemented a bootstrap aggregating procedure [33] to handle the highly unbalanced large training datasets. A developer can easily define/incorporate a new classifier such as random forest [34] by implementing the BinaryClassifier interface and integrate it into the bootstrap aggregating procedure and/or the machine learning framework.

The feature extraction module defines the features to be extracted from data and used in a classifier. Currently, we have integrated three sets of features: *k*-nearest neighbor (KNN) scores, protein disorder scores, and amino acid (AA) frequencies. A developer can incorporate new features into Musite simply by implementing the FeatureExtractor interface.

The training and prediction module defines the machine learning procedure by utilizing the classifier module and feature extraction module. MusiteTrain defines the model training procedure. It extracts features from training sequences and trains prediction models using the extracted features. MusiteClassify defines the prediction procedure. It extracts features from query sequences (new sequences from users for prediction) and makes predictions using the extracted features based on some prediction model trained by MusiteTrain.

The I/O module provides utilities for reading/writing different types of files and converting between them. Currently Musite supports file formats of FAST A, Musite XML, UniProt XML, and Phospho.ELM report. To support other file formats, a developer can implement the Reader and/or Writer interfaces. The MusiteIOUtil class provides uniform methods to access different Readers /Writers.

The UI module provides users with a biologist-friendly graphical user interface (GUI) to most functionalities in Musite. With the GUI, one can easily perform phosphorylation-site prediction, result analysis, stringency adjustment, customized model training, prediction model management, file format conversion, etc.

## Results and discussion

### Open framework for phosphorylation-site prediction

With its extensible API, Musite provides an open framework for phosphorylation-site prediction. With the rapidly accumulating data and better understanding of protein phosphorylation over time, more evidence needs to be integrated for better prediction performance. Musite provides a platform for integration of increasingly more diverse data and knowledge on protein phosphorylation. For example, phosphoproteomics data are scattered among different web resources. Musite can already read different file formats and can be easily extended to support more. It also provides functionality of merging phosphorylation annotations from different sources. Moreover, Musite makes it simpler to incorporate more biological evidence as features for phosphorylation-site prediction. With the open framework of Musite, it is possible to build a community-based tool, which could integrate the different expertise of various people from diverse areas and coordinate a joint effort towards better prediction and understanding of protein phosphorylation.

### Better utilization of the large magnitude of data

One challenge in phosphorylation-site prediction, similar to many other bioinformatics problems, is how to handle the magnitude of data. There are two issues: 1) how to utilize the large amount of experimentally verified phosphorylation data; 2) how to perform proteome-scale applications. Musite’s I/O module and its associated XML format provide a solution for collecting phosphorylation data from various sources. The bootstrap aggregating (bagging) procedure [33] implemented in Musite provides a solution for utilization of large datasets in machine learning applications and it also solves the problem of highly unbalanced data between positive and negative data. This procedure samples representative small datasets from large unbalanced datasets for training prediction models and aggregates prediction results from multiple classifiers for more robust performance. At the application level, Musite, as standalone software, can perform phosphorylation-site prediction up to the proteome scale on personal computers in an automated fashion. Moreover, users can utilize the customized model training utility to take advantage of the latest phosphorylation data.

### Integration into experimental design

Musite is a good candidate for integration into experimental studies because of its two unique utilities: customized model training and continuous stringency adjustment. Customized model training enables the users to train their own models from any phosphorylation dataset. Continuous stringency adjustment makes it possible for users to choose any stringency to meet their requirements for confidence level. Using these two utilities, Musite can be integrated into experiments for more efficient identification of phosphorylation sites. For example, in a hypothesis-driven experiment, after an experimental biologist gets some initial phosphorylation data from experiments, he or she could make proteome-scale predictions based on the initial dataset, and then focus more on predictions above a certain confidence level (using stringency adjustment) in the next stage; after each stage, the prediction model can then be refined (using customized model training) based on the new data and guide the experiments in the next stages. Using such an iterative design combining experimental and computational approaches, phosphorylation site identification could be much more efficient and less expensive.

### Case study: training an AIDS-specific model using Musite

A common limitation of all phosphorylation-site prediction tools is that prediction results cannot be correlated with different cell states or tissue conditions. Similarly, prediction results based on pre-trained models released in Musite 1.0 may only indicate whether a query site can be phosphorylated or not, but have no implications for cell types or states. However, it is possible to train tissue- or disease-specific models using the customized model training utility in Musite. In this section, a sample recipe of training an AIDS-specific phosphoserine/threonine prediction model is provided as follows.

• Step 1: retrieve AIDS-specific protein data from UniProt.

i. Open http://www.uniprot.org and search for *keyword:aids AND* reviewed:yes.

ii. Download the *complete data in XML format*.

• Step 2: Convert the downloaded UniProt XML file to the Musite XML format.

i. Start Musite.

ii. Open menu *Tools => File Processing => File Conversion => Convert UniProt XML to Musite XML.*

iii. Select the downloaded file as *from* file, and specify a *save to* file,

iv. Select all checkboxes in the *Site annotation status* section.

v. Click *OK* and wait until it is finished.

• Step 3 (optional): Check the abundance of phosphorylation sites

i. Open menu *Tools =>Statistics=>Sites Statistics*.

ii. Select the converted Musite XML file,

iii. Select *Phosphorylation as PTM type*.

iv. As of September 27, 2010, there were 504 proteins in the Musite XML file, out of which 248 were phosphoproteins, and there were 423 phosphoserines and 113 phosphothreonines.

• Step 4: Predict disorder scores.

i. Open menu *Tools => Feature Extraction => Disorder Prediction*.

ii. Select the converted Musite XML file as the sequence file, and specify a *save* to file.

iii. Click *OK* and wait until it is finished.

• Step 5: Train a prediction model.

i. Open menu *Tools => Prediction Model Training*.

ii. Select the Musite XML file with the disorder scores saved in the last step.

iii. Select *Serine(S)* and *Threonine(T)* as Residue Types.

iv. Click *OK* and wait until it is finished.

• Step6 (optional): Edit the trained model.

i. Open menu File =>*Manage Trained Model.*

ii. Double click on the model file just trained,

iii. Right click on the model to rename,

iv. Add comments and save.

• The trained model is ready for phosphorylation-site prediction from query sequences.

## Conclusions

With the rapidly accumulating phosphoproteomics data in recent years, the area of phosphorylation-site prediction has attracted increasingly more interest and attention. Musite provides an open-source framework for easy integration of new evidence and/or methodologies for better phosphorylation-site prediction. By providing an open resource for protein phosphorylation research, we hope that Musite could eventually evolve into a joint effort in the phosphorylation research community for both bioinformaticians and biologists. We are also expanding the scope of Musite to predict other types of PTM sites, such as acyletation, ubiquitination, protein methylation, and tyrosine sulfation.

## Availability and requirements

**• Project name:** Musite

**• Project home page:**http://musite.sourceforge.net

**• Operating system(s):** Platform independent

**• Programming language:** Java

**• Other requirements:** Java 5 or higher

**• License:** GNU GPL V3

**• Any restrictions to use by non-academics:** Musite has no general restriction to use by non-academics. However, Musite integrates SVM^light^ (http://www.cs.cornell.edu/People/tj/svm_light/) and VSL2 (http://www.ist.temple.edu/disprot/Predictors.html), which require permissions from their authors for commercial use.

## List of abbreviations

AA: amino acid; API: application programming interface; GPL: general public license; GUI: graphical user interface; I/O: input/output; KNN: *k*-nearest neighbour; OSI: Open Source Initiative; PTM: posttranslational modification; XML: eXtensible Markup Language; SVM: support vector machine;

## Competing interests

None.

## Authors' contributions

DX conceived the project. DX and JG design the methodology. JG implemented the software. JG drafted the manuscript. Both authors read and approved the final manuscript.
